# Patient reported pain-related outcome measures after tonsil surgery: an analysis of 32,225 children from the National Tonsil Surgery Register in Sweden 2009–2016

**DOI:** 10.1007/s00405-017-4679-4

**Published:** 2017-08-16

**Authors:** Fredrik Alm, Joacim Stalfors, Pia Nerfeldt, Elisabeth Ericsson

**Affiliations:** 1 Department of Anaesthesia and Intensive Care, Faculty of Medicine and Health, School of Health Sciences, Örebro University, Örebro, Sweden; 2 Institute of Clinical Sciences, Sahlgrenska Academy, University of Gothenburg, Gothenburg, Sweden; 3 Sheikh Khalifa Medical City, Ajman, United Arab Emirates; 4 Department of Otorhinolaryngology, Karolinska University Hospital, Stockholm, Sweden; 5 Division of Clinical Science, Intervention and Technology, Karolinska Institute, Stockholm, Sweden; 6 School of Health Sciences, Faculty of Medicine and Health, Örebro University, Örebro, Sweden

**Keywords:** Children, Pain, PROM, Tonsillitis, Tonsillar hypertrophy, Tonsillectomy, Tonsillotomy

## Abstract

The objective of this study was to describe factors affecting pain after pediatric tonsil surgery, using patient reported pain-related outcome measures (pain-PROMs) from the National Tonsil Surgery Register in Sweden. In total, 32,225 tonsil surgeries on children (1 to <18 years) during 2009–2016 were included; 13,904 tonsillectomies with or without adenoidectomy (TE ± A), and 18,321 tonsillotomies with or without adenoidectomy (TT ± A). Adjustments were made for variables included in the register to compensate for contributable factors in the analysis. When compared to TE ± A for surgical indication obstruction, TT ± A resulted in lower pain-PROMs, shorter use of postoperative analgesics, earlier return to regular food intake, and lower risk for contact with health care services due to pain. Children who underwent TE ± A because of obstruction problems stopped taking painkillers and returned to normal eating habits sooner, compared to children who underwent TE ± A for infectious indications. In both indication groups, TE ± A performed with hot rather than cold technique (dissection and haemostasis) generally resulted in higher pain-PROMs. Older children reported more days on analgesics and a later return to regular food intake after TE ± A than younger ones. No clinically relevant difference between sexes was found. Between 2012 and 2016 (pre-and post-implementation of Swedish national guidelines for pain treatment), the mean duration of postoperative analgesic use had increased. In conclusion, TE ± A caused considerably higher ratings of pain-related outcome measures, compared to TT ± A. For TE ± A, cold surgical techniques (dissection and haemostasis) were superior to hot techniques in terms of pain-PROMs. Older children reported higher pain-PROMs after TE ± A than younger ones.

## Introduction

Tonsil surgery with or without adenoidectomy is one of the most common surgical procedures performed on children worldwide [1, 2]. The most common indication is infectious (e.g., recurrent tonsillitis, chronic tonsillitis, and peritonsillar abscess), and upper airway obstruction due to hypertrophy of the lymphatic tissue in the Waldeyer’s ring [3, 4]. Traditionally, total tonsillectomies (TE) are performed on children for treatment of both types of indications. In the last decade, some countries have reported an increasing trend in partial tonsillectomy/tonsillotomy (TT) on children with tonsil-related upper airway obstruction, due to less postoperative morbidity compared to TE [4–6]. Tonsil surgery is a painful childhood surgical procedure that causes moderate to severe pain many days after the surgery [7–10]. When the risk for postoperative hemorrhage and infection is also included [11, 12], tonsil surgery can be considered to cause significant morbidity during recovery. In many countries, a majority of tonsil surgery procedures are performed in outpatient settings [1, 6]. This means that the postoperative recovery takes place at home, with the children and caregivers being responsible for the pain and symptom management [13].

The National Tonsil Surgery Register in Sweden (NTSRS) is a quality assurance system for tonsil surgery. All Swedish ENT-clinics are encouraged to submit data. The aim of the register is to give feedback on the outcome of the surgical procedure to facilitate quality improvements. In 2013, register data demonstrated a high number of patient reported contacts with health care services due to pain. For quality improvement purposes, a national survey was conducted by the NTSRS. It revealed a lack of consensus on pain management, insufficient pharmacological treatment, and lacking patient-centered information. The survey identified the necessity of better evidence-based pain treatment guidelines for pediatric tonsil surgery [14]. This resulted in the development and implementation of the Swedish national guidelines from NTSRS, together with updated patient information published in several languages on the website http://www.tonsilloperation.se. The Swedish national guidelines recommend multimodal pain treatment. Premedication is the start of the multimodal pain approach and includes oral paracetamol (acetaminophen), clonidine and betamethasone. After discharge from hospital, the recommendations for pain relief are paracetamol combined with COX-inhibitors (ibuprofen or diclofenac) and, if needed, oral clonidine rather than opioids (rescue analgesics). According to the guidelines and the website http://www.tonsilloperation.se, analgesic treatment after tonsillectomy is usually required for 5–8 days, and 3–5 days after tonsillotomy. Parents are recommended to contact the hospital if the child has difficulties to drink or eat adequately or suffers from pain despite taking the recommended medication [15].

To further improve care for pediatric patients and give evidence-based advice there is a need to identify how factors affect the outcome. The NTSRS collects perioperative data from health care professionals and patient reported outcome measures 30 days and 6 months, respectively, after surgery. This provides an opportunity to explore how surgical methods (i.e., TE or TT), surgical indication, age, sex, and surgical techniques (dissection and haemostasis) affect postoperative pain. Pain is evaluated as number of days on analgesics, number of days to regular food intake, and frequency of contacts with health care services due to pain. The objective of this study was to describe the factors affecting pain after pediatric tonsil surgery, using patient reported pain-related outcome measures from the National Tonsil Surgery Register in Sweden.

## Method

### Study design

A retrospective cohort study with prospective data inclusion, based on data from the National Tonsil Surgery Register in Sweden.

### Register

The NTSRS includes patients with benign indications undergoing tonsillectomy with or without adenoidectomy (TE ± A), and tonsillotomy with or without adenoidectomy (TT ± A). Register data are recorded through questionnaires at several time points (Table 1). The first questionnaire is used to collect data on age, sex, social security number and indication, and is filled in preoperatively by the health care professionals. The second questionnaire is filled in by the professionals after the surgery. The data recorded concerns surgical method, dissection technique, haemostasis technique, postoperative bleeding, and actions taken against bleeding during the hospital stay. Thirty days after surgery, an e-mail with a link to a secure website is sent to the patient/caregivers requesting them to answer a questionnaire. If no e-mail address is available, the questionnaire is sent by regular mail together with a prepaid envelope. The questionnaire includes patient reported outcome measures (PROMs) about postoperative recovery, including pain, infection, hemorrhage, and how the patient information was perceived. A fourth questionnaire is sent to the patient/caregivers using the same procedure 6 months after surgery, to record data on symptom relief. The NTSRS coverage has been mapped against the Swedish National Patient Register, which is managed by The National Board of Health and Welfare, an agency of the Ministry of Health and Social Affairs. The mapping was performed by matching personal identity numbers. The coverage for the period 2009–2015 was 48.1, 63.2, 78.4, 75, 82.3, 81.6, and 83.2%.

**Table 1:** Summary of questions to collect data in the National Tonsil Surgery Registry in Sweden

1st Questionnaire (filled in by the professionals before surgery)
Age?
Sex?
Indication?
2nd Questionnaire (filled in by the surgeon before discharge from hospital)
Operating method?
Technique for dissection?
Technique for haemostasis?
Bleeding complications during hospital stay?
3rd Questionnaire (answered by the patient or caregivers 30 days after surgery)
How many days after the operation did you/your child take painkillers?^a^
How many days after the operation did you/your child begin to eat regular food?^b^
Have you contacted health care services due to pain after the operation?^c^
Did any infection occur during hospitalization or within 30 days after operation?
Have you contacted health care services due to infection?
Were you/your child prescribed antibiotics due to infection?
Have you contacted health care services because of bleeding from the throat?
Were you/your child readmitted to the hospital because of bleeding from the throat?
Was surgery performed to stop the bleeding?
Did the information you/your child received correspond to how you/your child experienced the surgery and the time afterwards?
Have you/your child studied the patient information on http://www.tonsilloperation.se?
4th Questionnaire (answered by the patient or caregivers 6 months after surgery)
Degree of symptom relief?

A full description of the questionnaires is available at the National Tonsil Surgery Register in Sweden [14]

^a, b, c^Patient reported pain-related outcome measures (pain-PROMs) that this study aims to evaluate

### Procedure

This study includes all children aged 1 to <18 years who received TT ± A or TE ± A for the indication upper airway obstruction or infection, and answered the 30-day questionnaire between the 1st of January 2009 and the 2nd of November 2016. The focus of the present study was patient reported pain-related outcome measures (pain-PROMs) from the 30-day patient questionnaire. These were; (a) How many days after the operation did you or your child take painkillers? (b) How many days after the surgery did your child begin to eat regular food? (c) Have you contacted health care services due to pain after the surgery? Question B was added in 2013 and was, therefore, evaluated in fewer patients.

Pain-PROMs was explored in three surgical method/indication groups: TT ± A for the indication obstruction, TE ± A for the indication obstruction, and TE ± A for the indication infection. The effects of age, sex, surgical technique for haemostasis and surgical technique for dissection were each evaluated on the three pain-PROMs in the three surgical method/indication groups. This resulted in nine analyses on each investigated factor; age, sex, surgical technique for dissection, and surgical technique for haemostasis.

The surgical techniques for haemostasis and dissection were categorized as cold and hot as follows: cold dissection technique (cold steel), cold haemostasis technique (none, infiltration with epinephrine, ties, suture ligature), hot dissection technique (radiofrequency, bipolar diathermy scissors, bipolar diathermy, ultracision), and hot haemostasis technique (unipolar diathermy, bipolar diathermy, radiofrequency).

To study differences in pain-PROMs data between pre- and post- implementation of the Swedish national guidelines for treatment of pain, data from 2012 and 2016 was compared.

### Ethics

The present study was approved by the Central Ethical Board in Uppsala (application number 2016/445).

### Statistical analysis

Continuous variables are described with mean, standard deviation (SD), median and interquartile (*q*1; *q*3). Categorical variables are presented with number and percentages. For comparisons between the two groups, Fisher’s exact test was used for dichotomous variables whereas Mann–Whitney *U* test was used for continuous variables. Mean difference and odds ratio are presented both unadjusted as well as adjusted for the following four variables; dissection technique (hot/cold), haemostasis technique (hot/cold), age and sex. When analyzing the effect of each individual variable, adjusted mean difference or odds ratio for the variables is presented, with adjustments made for the other three variables. Adjustments were conducted using covariance analysis for continuous variables and multivariable logistic regression for dichotomous variables. Spearman’s rank correlation (*r*_s_) was used to analyse correlation between two continuous variables. The tests were two-tailed and conducted at 1% significance level. Statistical analyses were performed using SAS version 9.4.

## Results

A total of 53,028 tonsil surgeries on children were reported to the NTSRS between the 1st of January 2009 and the 2nd of November 2016. Sixty percent (*n* = 32,225) of the patients/caregivers answered the questionnaire 30 days after surgery and were included in the present study (Table 2). A drop-out analysis showed no significant difference between non-respondents and participants regarding sex (53.4 vs 53.1% male, *p* = 0.47), whereas the respondents were about 2 months older (mean age 7.14 vs 6.99, *p* = 0.0096).

**Table 2:** Demographic data and surgical technique for the total cohort and each surgical method/indication group. Comparison of sex, age, cold/hot dissection and haemostasis for TT ± A obstruction versus TE ± A obstruction, and TE ± A obstruction versus TE ± A infection

Method indication	Total (*n* = 32,225)	TT ± A obstruction (*n* = 18,109)	TE ± A obstruction (*n* = 7204)	TE ± A infection (*n* = 6700)	TT ± A obstruction versus TE ± A obstruction (*p* value)	TE ± A obstruction versus TE ± A infection (*p* value)
Sex						
Male	17,117 (53.1%)	10,291 (56.8%)	3915 (54.3%)	2811 (42.0%)		
Female	15,108 (46.9%)	7818 (43.2%)	3289 (45.7%)	3889 (58.0%)	0.0003	<0.0001
Age						
Mean (SD)	7.14 (4.57)	5.37 (2.90)	7.22 (4.53)	11.8 (5.0)		
Median	5.52	4.59	5.54	13.0		
Min; max	1.04; 18.0	1.04; 17.98	1.09; 18:00	1.4; 18:0	<0.0001	<0.0001
Technique for dissection^a^						
Cold	10,729 (34.2%)	462 (2.6%)	5485 (79.0%)	4753 (73.3%)		
Hot	20,681 (65.8%)	17,315 (97.4%)	1458 (21.0%)	1732 (26.7%)	<0.0001	<0.0001
Technique for haemostasis^b^						
Cold	9503 (32.0%)	6884 (42.8%)	1456 (20.9%)	1102 (17.1%)		
Hot	20,162 (68.0%)	9205 (57.2%)	5504 (79.1%)	5334 (82.9%)	<0.0001	< 0.0001

*SD* standard deviation, *n* number of responds on 30-day survey, *TE* *±* *A* tonsillectomy with or without adenoidectomy, *TT* *±* *A* tonsillotomy with or without adenoidectomy

^a^Cold dissection technique: cold steel. Hot dissection technique: radiofrequency, bipolar diathermy scissors, bipolar diathermy, ultracision and multiple techniques where one or more techniques were hot. Missing value *n* = 815

^b^Cold haemostasis technique: none, infiltration with epinephrine, ties, suture ligature. Hot haemostasis technique: unipolar diathermy, bipolar diathermy, radiofrequency and multiple techniques where one or more techniques were hot. Missing value *n* = 2560. TT ± A indication infection group (*n* = 212) is only presented in total and not in a separate group

The children who had tonsil surgery due to obstruction were younger (mean age 5.89 vs 11.7, *p* < 0.0001) and predominately male (56.1 vs. 42.1% female, *p* < 0.0001), compared to the children who had tonsil surgery for infectious indications. The majority of the children operated for obstructive symptoms underwent TT ± A (71.5%), compared to TE ± A (28.5%). TE ± A was most often performed with cold dissection techniques, while hot dissection techniques were the most common ones used for TT ± A (Table 2).

### Grouping arrangements

To group the patient data, we analyzed if tonsil surgery (TT and TE) performed with or without adenoidectomy (±A) influenced the three pain-PROMs. Simultaneous adenoidectomy showed no significant differences after adjusting for surgical dissection technique, haemostasis technique, age and sex. Therefore, data are presented with or without adenoidectomy (±A) in the following surgical method/indication groups; TT ± A for indication obstruction (*n* = 18,109), TE ± A for indication obstruction (*n* = 7204), and TE ± A for indication infection (*n* = 6700) (Table 2). Children who underwent TT ± A for indication infection were few (*n* = 212). They are only presented in the total cohort and not analyzed as a separate group.

### Surgical method and indication

Table 3 illustrates the results for the number of days on postoperative analgesics, the number of days to regular food intake, and frequency of contacts with health care services due to pain in each surgical method/indication group. Children who received surgery for the indication obstruction stopped taking analgesics and returned to regular eating habits sooner after TT ± A compared to TE ± A (*p* < 0.0001), while the significantly longest duration on postoperative analgesics and the latest return to regular food intake were recorded in children who underwent TE ± A on infectious indications (*p* < 0.0001). After adjustments for dissection technique, haemostasis technique, age and sex, it was shown that children operated with TT ± A had 2.72 days less of postoperative analgesics (*p* < 0.0001) and returned to regular food intake 1.84 days sooner (*p* < 0.0001), compared to TE ± A for the indication obstruction. The difference between children who underwent TE ± A on infectious indication and TE ± A on indication obstruction was small but still significant after adjustments; 0.45 days longer use of postoperative analgesics (*p* < 0.0001) and 0.49 days longer time to regular food intake (*p* = 0.0028) for the infectious indication group. For the total cohort, days on postoperative analgesics and days to regular food intake correlates with each other (*r*_s_ = 0.41, *p* < 0.0001).

**Table 3:** Patient reported pain-related outcome measures (pain-PROMs) for the total cohort and each surgical method/indication group. Comparison of days with analgesics, days to regular food intake and contacts with health care services due to pain for TT ± A obstruction versus TE ± A obstruction, and TE ± A obstruction versus TE ± A infection

Method indication	Total (*n* = 32,225)	TT ± A obstruction (*n* = 18,109)	TE ± A obstruction (*n* = 7204)	TE ± A infection (*n* = 6700)	TT ± A obstruction versus TE ± A obstruction	TE ± A obstruction versus TE ± A infection
					*Unadjusted mean difference (CI), p value* Adjusted mean difference (CI), *p* value*	*Unadjusted mean difference (CI), p value* Adjusted mean difference (CI), *p* value*
(a) Number of days with analgesics after surgery						
Mean (SD)	6.11 (4.63)	4.64 (3.65)	7.34 (4.70)	8.72 (5.32)	*2.71* (*2.58; 2.83*)*, <0.0001*	*1.38* (*1.22; 1.56*), *<0.0001*
Median (*q*1; *q*3)	6 (2; 7)	4 (2; 7)	7 (4; 10)	9 (6; 12)	2.72 (2.51; 2.92), <0.0001*	0.45 (0.25; 0.64), <0.0001*
	*N* = 28,888	*N* = 16,130	*N* = 6487	*N* = 6083		
**(**b) Number of days to regular food intake after surgery^a^						
Mean (SD)	4.07 (3.08)	3.19 (3.14)	4.95 (4.11)	6.19 (4.44)	*1.76* (*1.55; 1.97*)*, <0.0001*	*1.24* (*0.96; 1.55*), *<0.0001*
Median (*q*1; *q*3)	3 (1; 5)	2 (1; 4)	4 (2; 7)	5 (3; 8)	1.84 (1.53; 2.14), <0.0001*	0.49 (0.15; 0.83), 0.0028*
	*N* = 8922	*N* = 5629	*N* = 1593	*N* = 1646		
(c) Contacts with health care services due to pain after surgery						
Contacts (%)	3610 (12.2%)	1035 (6.2%)	1155 (17.5%)	1394 (22.8%)	*11.3%* (*10.3;12.3%*), *<0.0001*	*5.3%* (*3.9; 6.7%*), *<0.0001*
	*N* = 29,970	*N* = 16,695	*N* = 6589	*N* = 6114	–	–

*n* number of responds on 30-day survey, *N* number of respondents who answered the question, *SD* standard deviation, *CI* 95% confidence interval (bootstrapped), *TE* *±* *A* tonsillectomy with or without adenoidectomy, *TT* *±* *A* tonsillotomy with or without adenoidectomy

* Adjusted for operation technique, haemostasis technique, age and sex

^a^Outcome variable B, has been recorded in the register since 2013. TT ± A indication infection group (*n* = 212) is only presented in total and not as a separate surgical method/indication group

The frequency of contacts with health care services due to pain was also higher in the children who received TE ± A on indication obstruction, compared to TT ± A (17.5 vs 6.2%, *p* < 0.0001). The significantly highest frequency of contacts was presented in children who underwent TE ± A on infectious indications (22.8%, *p* < 0.0001).

### Surgical technique for dissection and haemostasis

**Fig. 1:**
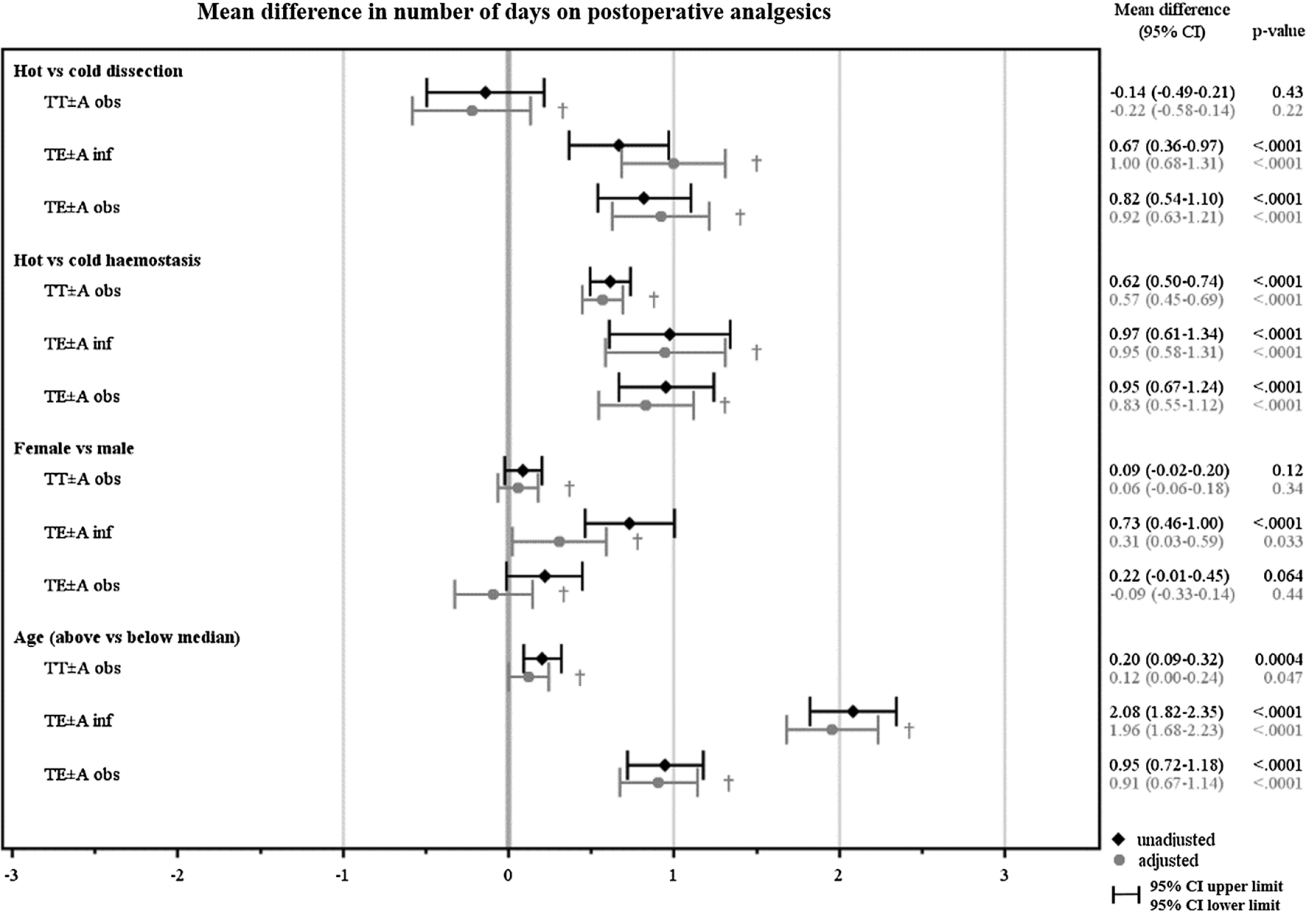
Illustrates the unadjusted and adjusted mean difference, and 95% confidence interval (CI) in the number of days on postoperative analgesics between hot vs cold dissection technique, hot vs cold haemostasis technique, female vs male and age above vs below median for each surgical method indication group. † Adjusted mean difference for each of the four variables (dissection technique, haemostasis technique, sex and age) is presented, with adjustments made for the other three variables

**Fig. 2:**
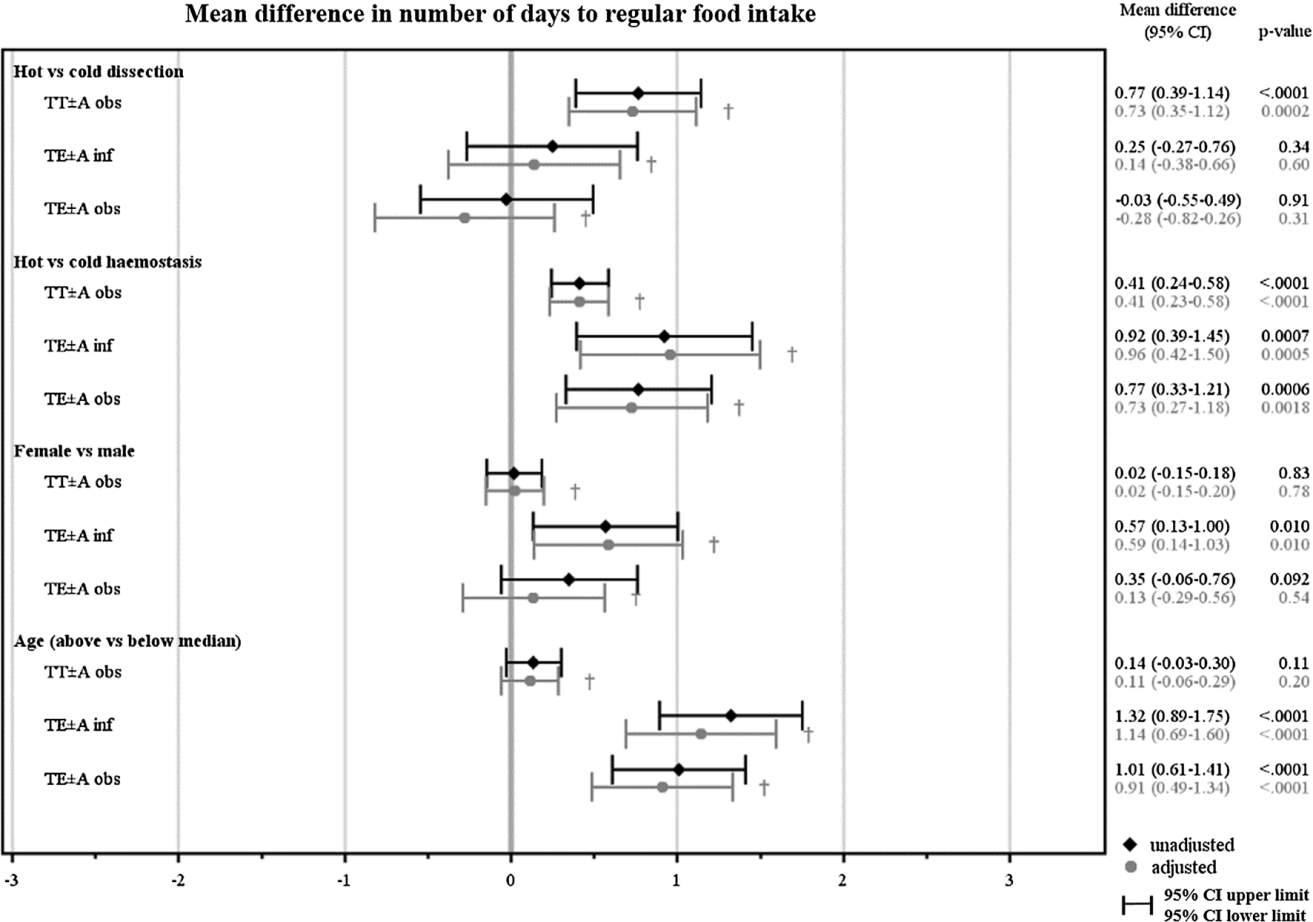
Illustrates the unadjusted and adjusted mean difference and 95% confidence interval (CI) in number of days to regular eating habits between hot vs cold dissection technique, hot vs cold haemostasis technique, female vs male and age above vs below median for each surgical method indication group. † Adjusted mean difference for each of the four variables (dissection technique, haemostasis technique, sex and age) is presented, with adjustments made for the other three variables

**Fig. 3:**
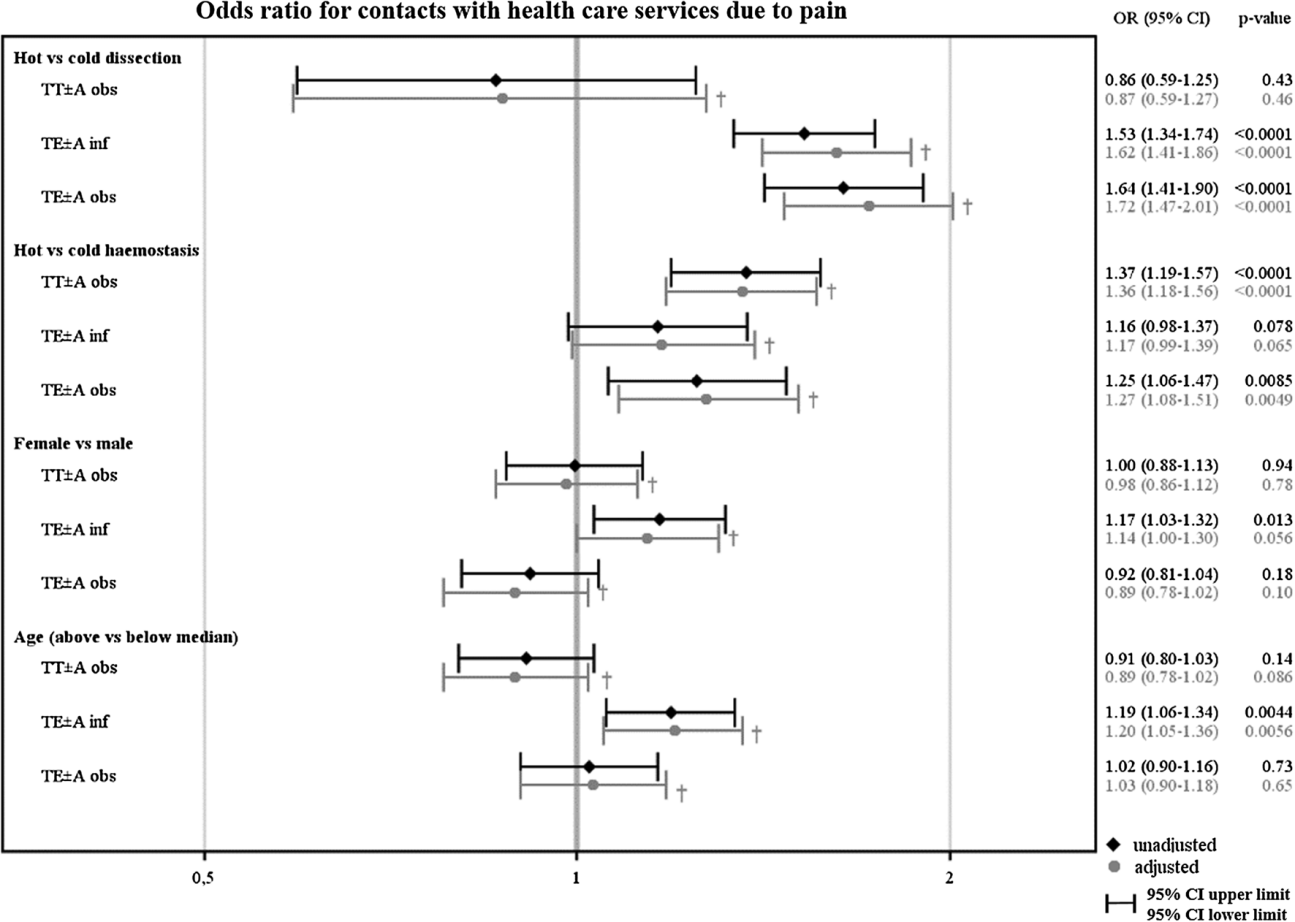
For each surgical method indication group, unadjusted and adjusted odds ratio (OR) and 95% interval (CI) for contacts with health care service due to pain after surgery are presented for hot vs cold dissection technique, hot vs cold haemostasis technique, female vs male, and age above vs below median. † Adjusted odds ratio for each of the four variables (dissection technique, haemostasis technique, sex and age) is presented, with adjustments made for the other three variables

After adjustment, TE ± A performed with hot rather than cold dissection technique resulted in approximately 1 day longer use of postoperative analgesics in both children operated on indication infection (*p* < 0.0001) and obstruction (*p* < 0.0001). After adjustments, hot dissection technique was still a risk factor for contacts with health care services due to pain in both children operated on indication infection (OR 1.62, *p* < 0.0001) and obstruction (OR 1.72, *p* < 0.0001). Altogether, before and after adjustment for haemostasis technique, age and sex, hot dissection technique presented a significantly higher outcome in five of nine analyses than cold technique (3 groups × 3 pain-PROMs = 9 analyses), see Figs. 1, 2 and 3.

After adjustment, hot haemostasis technique resulted in approximately 1 day longer use of postoperative analgesics after TE ± A both in children operated on indication infection (*p* < 0.0001) and obstruction (*p* < 0.0001), and about half a day longer analgesic use after TT ± A (*p* < 0.0001), compared to cold technique. Altogether, before and after adjustment for dissection technique, age and sex, hot haemostasis technique presented a significantly higher outcome in eight of nine analyses than cold technique (3 groups × 3 pain-PROMs = 9 analyses), see Figs. 1, 2 and 3.

### Sex and age

Sex did not influence pain outcomes. There were no significant differences in pain-PROMs between sexes after adjustments for dissection technique, haemostasis technique and age, with the single exception in TE ± A indication infection group where days to regular food intake was somewhat later in the girls (mean difference 0.59 days, *p* = 0.01; Fig. 2).

Age was dichotomized to younger children (<median years of age) or older children (≥median years of age), guided by the median age of each surgical method/indication group. Older children took postoperative analgesics approximately 2 days longer (*p* < 0.0001) in the TE ± A indication infection group (*p* < 0.0001), and 1 day longer in the TE ± A indication obstruction group (*p* < 0.0001). Furthermore, older children returned to regular food intake about 1 day later after TE ± A in both indication groups (*p* < 0.0001). After adjustment, older children presented a significantly higher outcome in five of nine analyses (3 groups × 3 pain-PROMs = 9 analyses), compared to younger children, see Figs. 1, 2 and 3.

### Postoperative infection

In the total cohort, the children (or their caregivers) who stated that they had contacted health care services due to infection within 30 days after surgery used postoperative analgesics about 2 days longer [mean difference 2.20 (95% CI 2.00; 2.40), *p* < 0.0001], and returned to regular food intake about 1 day later [mean difference 1.04 (95% CI 0.70; 1.38), *p* < 0.0001], compared to those who did not contact health care services due to infection. Significant differences were also observed when analyses were done on each surgical method/indication group.

### Swedish national guidelines for pain treatment and patient information at http://www.tonsilloperation.se

**Fig. 4:**
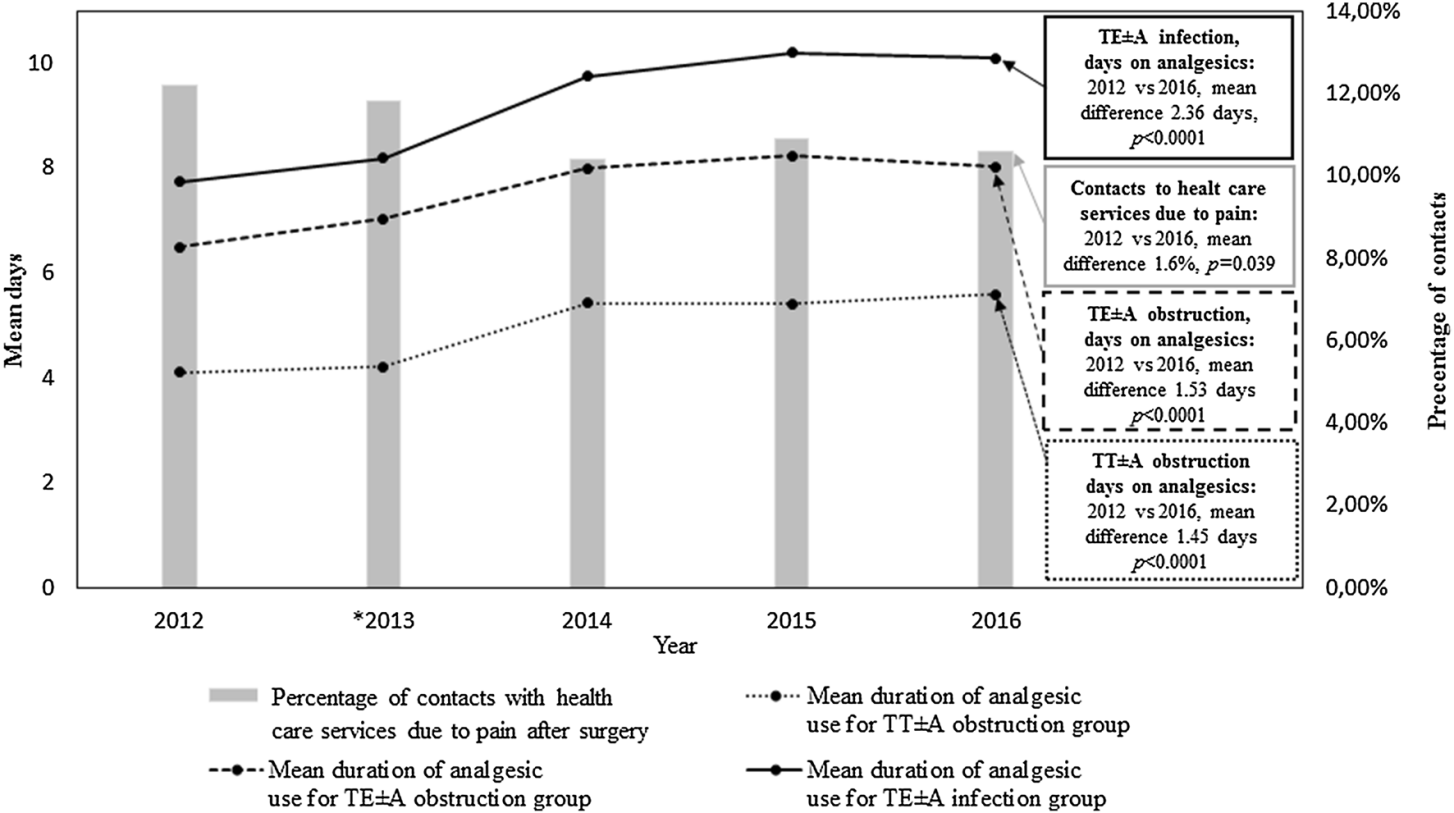
The *line graph* illustrates the mean duration in number of days with postoperative analgesics in 2012, 2013, 2014, 2015 and 2016 for each surgical method/indication group. The *bar graph* illustrates the percentage of contacts with health care services due to pain in 2012, 2013, 2014, 2015, and 2016. * The Swedish National guidelines for pain treatment were implemented in 2013, together with tailored patient information on the website http://www.tonsilloperation.se

Between 2012 and 2016 (pre- and post-implementation of Swedish national guidelines for pain treatment), the mean duration of postoperative analgesic use increased in each surgical method/indication group (*p* < 0.0001; Fig. 4). There was no significant difference between 2012 and 2016 in the frequency of contacts with health care services due to pain (*p* = 0.039; Fig. 4). Pain-PROMs “number of days to regular food intake” was first registered in the NTSRS in 2013 and was excluded from the analysis.

In the total cohort, the children (or their caregivers) who studied the patient information on the website http://www.tonsilloperation.se used postoperative analgesics longer (6.55 vs 6.06 mean days, *p* < 0.0001), and contacted health care services due to pain more frequently (12.7 vs 9.8%, *p* < 0.0001), compared to children/caregivers who did not visit the website. The significant differences were similar in each surgical method/indication group.

## Discussion

This study shows that there is significant pain during the postoperative recovery after tonsil surgery, affecting days on analgesics, children’s ability to eat regular food, and the number of contacts with health care services. In addition, the amount of data allowed for statistical analysis showing pain outcome depends on the surgical method and technique, as well as patient factors such as age and surgical indication. The implementation of the Swedish national guidelines and the targeted information on the website seem to have contributed to more days on analgesic treatment during the postoperative recovery.

The choice of operation method, TT ± A or TE ± A, is in focus in many countries. In Sweden, analyses from the NTSRS have shown that since 2011, TT ± A is more common than TE ± A when operating pediatric tonsils due to tonsillar hypertrophy. Furthermore, an overall increasing frequency of tonsil surgeries in children has been demonstrated, especially those performed for the indication obstruction [4]. This study shows that TE ± A is favored for infectious reasons, whereas both TT ± A and TE ± A are used for obstructive indications, with a significantly higher pain-PROMs after TE ± A.

Based on our register study, the TT ± A–group demonstrated less morbidity compared to TE ± A, with 3 days less on analgesics and a return to normal eating habits 2 days sooner. The results are comparable to a systematic review with randomized controlled trials comparing TT with TE [6]. In addition, a new study covering different aspects of postoperative recovery after tonsil surgery has demonstrated the advantages of TT ± A over TE ± A [16]. Faster return to regular eating habits and shorter period of pain treatment after TT are signs of faster healing. Surgery on the tonsils and the highly sensitive innervated mucosa and underlying muscle is painful, especially if the capsule is broken and the muscle is exposed [2], for instance, in cases of tissue damage following TE.

When choosing the operation method (i.e., TE ± A or TT ± A) for the indication obstruction, there are other aspects to consider beside postoperative pain. One is treatment success. The NTRS has earlier evaluated patient reported symptom relief for obstructive indications and found similar figures of improvement or relief in both TE and TT [17]. Few studies have evaluated the objective polysomnographic (PGS) outcomes after TT versus TE in a randomized setting in obstructive sleep apnea (OSA). A recent study on this topic found TT + A to be non-inferior to TE + A regarding reduction of apnea–hypopnea-index-scores and other PSG variables such as oxygen-desaturation-index, as well as quality of life questionnaire scores (OSA-18) [18]. Perioperative bleeding is another factor to consider. In a recent review, the risk of postoperative bleeding after TE was assessed, estimating the bleeding frequency after TE (again performed on indication OSA) in children to be 0.3% primary bleeding and 2.2% secondary bleeding, with a re-operation rate of 1.3%. This is approximately twice as high as the figures presented after TT; 1.3% primary bleeding and 0.3% secondary bleeding, with 0.6% re-operation rate [11]. The risk of bleeding seems to increase with age [19]. The risk of re-operation due to tonsillar re-growth is also a factor to consider. This has been estimated to be seven times higher after TT than TE, with the highest risk in the youngest children under the age of three and decreasing markedly with age [20]. Another recent systematic review found a re-growth rate of 6%, and 3% ultimately underwent revision surgery [21]. A final aspect is that even small differences in postoperative pain can have an impact on health care costs. The indirect cost of TE ± A has been estimated to be 61% higher than that of TT ± A [22]. TT-parents have to take less time off work after their child’s surgery, compared to TE-parents. According to Sathe et al. [20], RCT-data does not show that partial tonsillectomy markedly affects outcomes compared to total tonsillectomy. We would like to specify that the pain, bleeding, and health economic aspects indicate that there are more advantages in connection with TT compared to TE. However, there is a risk of re-growth after TT, especially in the youngest children. On the other hand, from a pain perspective, the majority of children undergoing TT does not need a re-operation and have a favorable postoperative recovery compared to children undergoing TE.

Higher pain-PROMs in the children who received surgery on indication infection compared to indication obstruction cannot only be explained by the difference in age at the time of surgery, as significant differences still existed after adjustments for age, sex and surgical techniques. The children with a history of repeated tonsil infection developed an inflammatory scar tissue, which may have mediated the effect on postoperative pain. The influence of surgical indication on post-TE ± A morbidity has been studied before in the NTSRS, revealing 1.23 day longer postoperative analgesic use in children with infectious indication [19]. However, differences between the indication groups have been clarified in the present study by showing the adjusted difference in number of days (0.45 days) on analgesics and number of days (0.49 days) to regular food intake. This constitutes only a minor clinical difference between the groups (approximately half a day).

Cold dissection technique causes less tissue damage compared to electrocautery [23], and convincing evidence suggests that TE performed with cold dissection techniques is superior to hot techniques in terms of postoperative late bleeding and pain [24–31]. The large sample size in the present study supports this by demonstrating longer time on analgesics and higher frequency of contacts with health care services in children operated with hot dissection technique. Beside dissection technique, cold rather than hot haemostasis technique seems to affect the pain-PROMs, with shorter use of analgesics and faster return to normal eating habits after both TE and TT.

Children/caregivers who reported that they had been in contact with the health care services due to infection within 30 days after operation also reported longer analgesic use and a later return to regular food intake. Routine treatment with antibiotics has generally shown no or small benefits in pain, diet or activity [32, 33]. In Sweden, antibiotics are not routinely prescribed, except for children with heart disease or other recognized reasons. Postoperatively, antibiotics are prescribed in about 10% [14]. The variable “contact with health care services due to infection” was not adopted in the adjusting analyses due to uncertain time frames and answers, depending on the children’s or caregivers’ subjective assessment of the infection status.

This study is unique as adjusted analyses have clearly demonstrated that there is minimal support for differences between the sexes with respect to pain-PROMs. Any clinical experience of more pain in girls probably depends on the fact that girls more often have surgery for the indication infection, compared to boys. They also tend to be older at the time of surgery, and these are two factors shown to influence pain-PROMs in the present study. There is limited research about sex differences after tonsil surgery. Some studies have proposed no sex differences in pain [34–36], while one postoperative tonsil recovery study in children showed a longer recovery process and remaining physical symptoms in girls [16].

The finding that older children generally report higher pain-PROMs after TE ± A is in accordance with previous research showing that older children and adults experience more postoperative pain after TE than younger children [16, 34–38]. As already stated, pain-PROMs is influenced by surgical indication. This needs to be taken into account in the age analyses as children having surgery due to indication infection tend to be older. In the present study this was considered by performing the age analyses in each surgical method/indication group. On the other hand, there were no differences in pain-PROMs between older and younger children undergoing TT. However, this finding should be interpreted with caution as the dichotomization of younger and older children varied between the surgical method/indication groups.

In all surgical methods/indication groups, days on analgesics has increased in the last 5 years. As recommended by the national guidelines and advised on the website http://www.tonsilloperation.se, analgesics is needed 5–8 days (sometimes longer) after TE ± A and 3–5 days after TT ± A [15]. It can be assumed that the information has reached patients and ENT-professionals and contributed to this change. The pain lasts for a long time after surgery and the increased use of analgesics should be seen as an improvement, where children’s pain is treated with a lower risk of pain-related complications such as sleep disturbance [10, 13], dehydration due to poor oral intake [10, 12, 13, 39, 40], and behavioral changes in the child [8, 10].

Contacts with health care services due to pain were common after both surgical methods (i.e., TE ± A and TT ± A). Notably, they were three times more frequent after TE ± A, illustrating the need to improve pain management, especially after TE ± A. In addition to paracetamol and cox-inhibitors (ibuprofen/diclofenac), the national guidelines recommend additional rescue analgesics with clonidine or opioids [15]. Despite this recommendation, a recently completed survey showed that only half of Swedish clinics prescribe rescue analgesics after TE ± A [41]. Furthermore, after TE ± A, children returned to normal eating habits later compared to TT ± A. Pain is the main reason for poor oral intake after tonsil surgery and there are children who lose a significant part of their preoperative weight [40]. To avoid a general catabolic state, optimal analgesics are important from the start and not after the patient have contacted the health care services. One study indicated that a majority of the children who were supplied with rescue analgesics after TE ± A used it. Most commonly, the first dose was taken on postoperative day 3 [42]. Increased knowledge about rescue analgesics is needed among health care professionals, children and their caregivers.

Children are not able to control the pain treatment or report the outcome to NTSRS on their own. This could be problematic and studies have shown that parents underestimate the pain [43]. Still, the NTSRS questionnaire that is sent to caregivers contains objective questions about, for instance, days to normal food intake instead of parents’ subjective assessment of the child’s pain by means of a visual analog scale. Days on analgesics and contact with health care services are also closely related to pain after surgery. When comparing the surgical indication/method groups, pain-PROMs correlate with each other. The group with the longest analgesic treatment also has most days to regular food intake and more contacts with health care services. When analyzing the factors age, sex, surgery technique for dissection and haemostasis, the outcome was not always simultaneously significant in all three Pain-PROMs. Therefore, the factors’ impact on pain should be interpreted on the basis of an overall picture of all results. To cover the entire pain situation, more items related to recovery are needed. One suggestion is the recently validated self-reported questionnaire Postoperative Recovery in Children (PRiC), which contains 21 items concerning different aspects of recovery after tonsil surgery, using text and photo illustrations [44]. PROMs is the golden standard for the planning and follow-up of delivered care, which should also be an axiom for children.

## Limitations

The data used for the present study were obtained from The National Quality Register in Sweden. Between 2009 and 2015 the national coverage increased from 48 to 83%, while the patient questionnaire response was 62% for the study period 2009–2016. The large coverage and the large population (*n* = 32,225) ensure external validity. Preferably, the response rate for the questionnaire would be higher, and this could cause a bias in the reporting of pain-related outcomes. Non-respondents to the questionnaire 30 days after surgery did not differ in sex and were only a mean of 2 months older. Furthermore, another study has matched NTSRS data regarding postoperative hemorrhage with data from the National Patient Register (NPR) in Sweden [30]. Reassuringly, there were no signs of neither over- nor under reporting of postoperative bleeding in the NTSRS. Thus, the rates in the questionnaires should be trustworthy.

Due to limitations in data, a register study is not the recommended study design for studying causalities, as it is mainly used for studying risk factors. A national register with a large coverage and many patients makes it possible to adjust for indicators included in the register and thus compensate for contributable factors in the analysis and present risk factors adjusted to these. This is obviously a great advantage, but it also involves the obvious disadvantage of not being able to adjust for the potential indictors not included in the register.

## Conclusion

The results of this study demonstrate that pain after pediatric tonsil surgery is complex, with several influencing factors. TE ± A causes considerably higher ratings of pain-related outcome measures (pain-PROMs), compared to TT ± A. For TE ± A, cold surgical techniques (dissection and haemostasis) are superior to hot techniques in terms of pain-PROMs. No clinically relevant difference between sexes was found. Older children reported higher pain-PROMs after TE ± A than younger ones. The implementation of the Swedish national guidelines and the targeted information on the website seem to have contributed to more days on analgesic treatment during the postoperative recovery. These findings reveal that particularly older children undergoing tonsillectomy have a need for improved analgesics for a better recovery. Pain management after tonsil surgery has been, and still is, a challenge for ENT-professionals.
